# Dignity and psychosocial related variables in elderly advanced cancer patients

**DOI:** 10.1186/s12877-022-03423-7

**Published:** 2022-09-05

**Authors:** Carla M. Martín-Abreu, Raquel Hernández, Patricia Cruz-Castellanos, Ana Fernández-Montes, David Lorente-Estellés, Helena López-Ceballos, Lorena Ostios-Garcia, Mónica Antoñanzas, Paula Jiménez-Fonseca, Teresa García-García, Caterina Calderon

**Affiliations:** 1 Department of Medical Oncology, Hospital Universitario de Canarias, Tenerife, Spain; 2 Department of Oncology Medical, Hospital Universitario La Paz, Madrid, Spain; 3 Department of Medical Oncology, Complejo Hospitalario Universitario de Ourense – CHUO, Ourense, Spain; 4 Department of Medical Oncology, Hospital Provincial de Castellón, Castelló de la Plana, Spain; 5 Department of Medical Oncology, Hospital San Pedro de Alcántara, Cáceres, Spain; 6 Department of Medical Oncology, Hospital Universitario Clínico San Carlos, Madrid, Spain; 7 Department of Medical Oncology, Hospital Universitario Central de Asturias, ISPA, Oviedo, Spain; 8 Department of Medical Oncology, Hospital Universitario Santa Lucia, Cartagena, Spain; 9 Department of Clinical Psychology and Psychobiology, University of Barcelona, Barcelona, Spain

**Keywords:** Dignity, Neoplasm, Anxiety, Social support, Functional limitations

## Abstract

**Introduction:**

Most cancers occur in older individuals, who are more vulnerable due to functional impairment, multiple comorbidities, cognitive impairment, and lack of socio-familial support. These can undermine patients’ sense of dignity. This study seeks to compare dignity scores in older patients with advanced cancer on sociodemographic and clinical variables and analyze the predictive value of anxiety, depression, functional limitations, and social support on dignity scores.

**Methods:**

A prospective, multicenter, observational study conducted with participation of 15 hospitals in Spain from February 2020 to October 2021. Patients with newly-diagnosed, advanced cancer completed the dignity (PPDS), anxiety and depression (BSI), Social Support (Duke–UNC-11), and functional limitations (EORTC-C30) scales. Lineal regression analyses explored the effects of anxiety, depression, functional status, and social support on dignity, adjusting for sociodemographic and clinical variables.

**Results:**

A total of 180 subjects participated in this study. The results of the correlation analysis revealed that dignity correlated negatively with anxiety, depression, and sex, and positively with social support, functional status, and longer estimated survival. Thus, women, and more anxious and depressed individuals scored lower on the dignity scale, whereas patients with more social support, fewer functional limitations, and longer estimated survival scored higher.

**Conclusion:**

In conclusion, being female, having a lower educational level, lower estimated survival, depression, anxiety, less social support, and limited functionality are correlated with less dignity in the elderly with advanced cancer. It is a priority to manage both physical and psychological symptoms in patients with unresectable advanced cancer to mitigate psychological distress and increase their sense of dignity.

## Introduction

The diagnosis of any disease provokes anxiety and uncertainty, but the diagnosis of cancer often involves psychological trauma and at times, social stigma. Cancer patients, especially those in advanced stages, must face tremendous physical and psychological changes that, in most cases, causes a state of vulnerability and dependence that can undermine their dignity [1]. There is a growing interest for older adults’ dignity in hospital care, long-term care, and end-of-life care [1, 2]. Dignity is an intrinsic, ascribed and dynamic quality of being human, demonstrates respect for self and others, and is associated with safe and peace [3]. The World Health Organization (WHO) emphasizes the need for a multidimensional approach that includes the psychological and spiritual needs of patients and their families. However, medical advances have not kept pace with the need to care for psychosocial or existential problems that are often neglected [2, 4]. Spiritual well-being can help individuals grapple with their illness, improve their quality of life, and protect them from end-of-life despair [5]. Knowing their prognosis; having family and social support, autonomy, hope, and meaning in life promote psychospiritual well-being and preservation of dignity [6]. Still, it must be remembered that each person is unique and each intervention must be tailored to their needs. Bovero et al. [7] reported that personality traits affect the loss of dignity and treatment should therefore be individualized.

Li et al. conducted a meta-analysis of 10 randomized controlled trials that compared therapy targeting dignity with standard care for patients with advanced cancer [4]. Dignity therapy was defined as individualized, short-term psychotherapy developed with the aim of relieving distress and improving end-of-life experiences of terminally ill patients and their families. They concluded that dignity therapies can be valuable in reducing anxiety, depression, and stress. Bauereiß et al. obtained similar results, which stresses the need for further studies to comprehend which specific interventions are effective and contribute to positive patient outcomes [5]. Chochinov et al. developed a study in which they recruited 50 terminal cancer patients and they conducted semi-estructurated interviewes to explore how participants coped with advanced illness and elaborate on their perceptions of dignity [8]. Finally, they reported that, since loss of dignity can increase depression, hopelessness, and the desire to hasten death, understanding the relationship between dignity and these psychosocial variables is pivotal for the integral management of the terminally ill, particularly in the case of hospitalized patients. More empirical research is requierd to establish and assess interventions that promote dignity and quality of life for people facing the end of life, inasmuch as understanding and promoting dignity affords the opportunity to respond with more sensitivity and determination to those nearing death.

More than 40% of cancers occur in the elderly (> 70 years), who are more vulnerable, due to functional decline, multiple comorbidities, cognitive impairment, and lack of socio-familial support [9]. This frailty entail greater complexity in the management of elderly age patients, such as receiving less aggressive treatment and less information about their disease [10]. However, several studies have found that younger patients have a more fractured sense of dignity [11–13]. Moreover, being elderly may actually be protective in coping with cancer, given these individuals’ acceptance of death and passive resignation [9].

To provide effective palliative care at the end of life, it is important to understand and identify the precipitating factors of emotional distress that result in a detriment to dignity. Hall et al. [14] conducted a study in which they described and compared the different sources of distress perceived by elderly cancer patients and those living in nursing homes. The sources of dignity-related distress were very similar in both groups, and they concluded that a better understanding of these would help clinicians provide more effective end-of-life care. On the other hand, Pergolizzi et al. [15] conducted a study comparing young and elderly people with advanced cancer. Older patients conveyed an apparent adaptation to dignity-related threats, especially in the domains of psychological and existential distress.

Despite the increased geriatric cancer population and the importance of understanding how dignity can influence their well-being, specific studies for this population are scarce. In this context, the primary objective of this study was to probe dignity scores in older patients with unresectable advanced cancer. The secondary objective was to examine how demographic, clinicopathological variables, anxiety, depression, functional limitations, and social support affect dignity scores. The hypothesis was that subjects with more social support, less anxiety and depression, and fewer functional limitations, would score better on the dignity scale.

## Methods

### Participants and procedures

NEOetic is a multi-institutional, prospective, observational study conducted with the participation of 12 hospitals in Spain between February 2020 and October 2021. The study was sponsored by the Bioethics Group of the Spanish Society of Medical Oncology (SEOM). Eligibility criteria included being 70 years of age or older with unresected, histologically confirmed, advanced cancer who were eligible for first-line systemic treatment. Of the 190 individuals enrolled, 180 were eligible and 10 were excluded (3 did not meet the inclusion criteria; 2 met an exclusion criterion and 5 had incomplete data).

The study was approved by the Spanish Agency of Medicines and Medical Devices (AEMPS) and the local research ethics committee of each hospital. Patients were invited to participate during their visit to the medical oncologist where a shared decision on systemic cancer treatment was made. All participants who agreed to take part signed written, informed consent forms. Participants completed the self-report form questionnaires at home and handed them in at the next visit to the study assistants. Clinical data were obtained from the medical records and all variables were collected on a website (http://www.neoetic.es).

### Measures (questionnaires)

Dignity was quantified using the Palliative Patient’s Dignity Scale (PPDS) [3]. The instrument consists of eight items, measuring the perception of dignity preservation, understood as being human and being self, feeling respected by others, respecting oneself, and preserving safety and peace; and threat to or loss of dignity, seen as feelings of insecurity and values violation, lack of support, or loss of feeling like “person”. Each response was scored on a Likert-type scale ranging from 0 (not at all) to 9 (very much), with total scores from 0 to 72. Higher scores indicate greater dignity. The value of Cronbach’s alpha was 0.75 [3].

Anxiety and depression were appraised using 12 of the 18 items of the Brief Symptom Inventory (BSI) [16] contemplated in the anxiety and depression subscales. The somatization subscale was not used as it could be confused with physical symptoms. These 12 items are divided into 2. 6-item subscales. The anxiety subscale examines nervousness, tension, motor restlessness, apprehension, and panic states, while the depression subscale gauges symptoms of disaffection and dysphoric mood, e.g., those reflecting worthlessness, anhedonia, hopelessness, and suicidal ideation. Each question is scored on a 5-Likert scale and each subscale score ranges from 0 to 24. Higher scores connote greater anxiety or depression. Raw scores are converted to T-scores, based on sex-specific normative data. The Spanish version of the BSI-18 has demonstrated good reliability and validity in Spanish patients [17]. Cronbach’s alpha scores for the anxiety and depression scales were 0.80 and 0.75, respectively [16].

Social support was assessed using the Duke–UNC-11 Functional Social Support Questionnaire [18]. This instrument explores two dimensions of social support: confidential support (received from people to whom the patient can communicate intimate feelings) and affective support (received from those who express positive empathy to patients). Each item is rates on a 5-point Likert scale, from 1 (much less than I would like) to 5 (as much as I would like). Scores range from 11 to 55; the higher the score, the more social support. Cronbach’s alpha was 0.93 [18].

Functional limitations were determined with the 15-item functional scale of European Organization for Research and Treatment of Cancer (EORTC) Quality of Life C-30 (QLQ-C30) Health-related quality of life (HRQoL) Questionnaire, version 3.0 [19]. The items comprise five multi-item functional scales: physical (PF), role (RF), cognitive (CF), emotional (EF), and social (SF). The questionnaire uses a 1-week time frame and responses are answered on a 4-point Likert-type scale (not at all, a little, quite a bit, and very much). All scores were linearly converted on a scale from 0 to 100, on which higher scores signify a higher level of functioning. In this sample, Cronbach’s alpha for the scale was 0.87.

Elixhauser Comorbidity Index was used to collect participants’ comorbidities based on International Classification of Diseases (ICD) diagnosis codes. The Eastern Cooperative Oncology Group (ECOG) scores describe a patient’s level of functioning in terms of their ability to care for themselves, their daily activity, and their physical ability (walking, working, etc.), ranging from 0 to 5, where 0 denotes perfect health and 5 denotes death.

### Statistical analyses

All statistical analyses were performed using the IBM-SPSS statistical package, version 25.0 for Windows (SPSS Inc., Chicago, IL). Count data were expressed by frequency and percentage (%); measurement data were reported as means and standard deviations, and independent sample t-test and ANOVA were used to compare psychological dignity scores across the study population’s different demographic characteristics. Pearson correlations analyses were conducted to analyze the correlation between variables. Multicollinearity among the variables was rejected by the variance inflation factor, which was < 5 for all and tolerance > 0.2 [20]. To ascertain the dignity, score predictive variable, a two-block linear regression model was performed. In the first block, anxiety, depression, functional scale, and social support were entered as criterion variables. In the second block, educational level, and survival were entered as independent variables, the analysis was performed for sex. We applied R-square and Cohen’s *f*^2^ standardized size measure to interpret the data [21]. For all analyses, significance was set at *p* < 0.05.

## Results

### Sociodemographic characteristics and their influence on dignity

A total of 180 patients participated in this study; 98 (54%) were males. Mean age was 76.0 years (SD = 4.5) and 24% (*n* = 73) were ≥ 80 years. Most were married or had a partner (69%) and had a primary level of education (62%). All the subjects were retired. As for clinical characteristics, 68% (*n* = 122) had more than four comorbidities according to the Elixhauser comorbidity index. The most common primary tumors were bronchopulmonary (28%), colorectal (16%), and pancreatic (17%). The most frequent histology was adenocarcinoma (64%). Nineteen percent of the participants had unresectable, locally advanced neoplasia and 81% had metastatic disease. Of the 73% who received chemotherapy as first-line systemic treatment, 10% were associated with targeted therapy and 6% with immunotherapy. Estimated survival was less than 18 months for 53% of the sample.

Of the 180 subjects, 45 patients scored low in dignity (percentile < 25; PD < 51), and 48 scored high in dignity (percentile> 75; PD > 65) on the PDDS scale. The mean dignity score was 57.9 (SD = 9.7). Table 1 illustrates the baseline characteristics of the sample and their influence on psychological dignity. Women, individuals with a primary-level education, and patients with lower estimated survival scored lower on the dignity scale than men, subjects with a higher level of education, and greater estimated survival (all *p* < 0.010). No significant intergroup differences were detected regarding the rest of the variables analyzed.

**Table 1:** Baseline characteristics and dignity score (*n* = 180)

Variables		n (%)	Dignity score	Statistics	
				t/f	***p***
**Sex**	Male	98 (54)	59.6 (8.7)	2.587	**0.010**
	Female	82 (46)	55.9 (10.5)		
**Age (years)**	70-80	137 (76)	57.8 (10.1)	−0.244	0.808
	> 80	73 (24)	58.2 (8.0)		
**Marital**	Married or partnered	146 (69)	57.8 (10.1)	0.208	0.836
	No partnered	34 (17)	57.2 (7.3)		
**Educational level**	Primary school or less	112 (62)	56.7 (9.8)	−2.152	**0.033**
	High school or greater	68 (38)	59.9 (9.4)		
**Tumor site**	Broncho-pulmonary	50 (28)	57.4 (11.1)	0.574	0.720
	Colorectal	28 (16)	59.1 (9.5)		
	Pancreas	30 (17)	56.2 (9.8)		
	Breast	4 (2)	54.0 (9.5)		
	Stomach	9 (5)	57.4 (9.8)		
	Others	59 (33)	59.0 (8.6)		
**Histology**	Adenocarcinoma	115 (64)	57.3 (10.1)	−1.130	0.260
	Others	65 (36)	59.0 (8.9)		
**Stage**	Locally advanced	35 (19)	57.9 (8.9)	−0.003	0.998
	IV	145 (81)	57.9 (9.9)		
**Estimated survival (months)**	< 18	96 (53)	56.5 (8.8)	−2.105	**0.037**
	≥18	84 (47)	59.5 (10.5)		
**Systemic treatment**	Chemoterapy (CT)	102 (57)	56.7 (9.6)	2.146	0.062
	CT + targeted therapy	18 (10)	61.1 (8.7)		
	CT + immunotherapy	11 (6)	63.7 (11.0)		
	Immunotherapy	20 (11)	54.9 (10.6)		
	Targeted therapy	9 (5)	61.2 (7.6)		
	Others	20 (11)	59.4 (9.0)		
**Elixhauser comorbidity index**	≤4	58 (32)	57.4 (9.8)	−0.481	0.631
	> 4	122 (68)	58.1 (9.7)		
**ECOG**	0-1	162 (27)	58.2 (9.8)	1.119	0.265
	2-3	18 (72.8)	55.5 (9.1)		

Bold values indicate significance at the 5% level

*Abbreviations:* Eastern Cooperative Oncology Group (ECOG)

### Correlations between variables and multiple linear regression analysis for dignity

The results of the correlation analysis in Table 2 revealed that dignity correlated negatively with anxiety, depression, and sex and positively with social support, functional status, and longer estimated survival. Thus, women and participants with more anxiety and depression had lower scores on the dignity scale, and individuals with more social support, fewer functional limitations, and longer estimated survival had scored higher.

**Table 2:** Pearson’s correlations between psychological, socio-demographic and clinical variables

Variables	1	2	3	4	5	6	7	
1. PPDS. Dignity	1							
2. BSI. Anxiety	−0.533^**^	1						
3. BSI. Depression	−0.591^**^	0.769**	1					
4. DUKE. Social Support	0.385^**^	−0.041	−0.142	1				
5. EORTC-QLQ-C30. Functional scale	0.589^**^	−0.560^**^	−0.586^**^	0.174*	1			
6. Sex: male	−0.190^*^	0.199^**^	0.258^**^	0.129	−0.205^**^	1		
7. Higher educational level	0.159^*^	−0.231^**^	−0.158^*^	− 0.047	0.196^**^	− 0.022	1	
8. Estimated survival ≥18 months	0.156^*^	−.022	−0.078	0.115	0.208^**^	−0.006	0.144	1

*Abbreviations*: *PPDS* Palliative Patient’s Dignity Scale, *BSI* Brief Symptom Inventory, *DUKE* Duke–UNC-11 Functional Social Support Questionnaire, *EORTC-QLQ-C30* European Organization for Research and Treatment of Cancer Quality of Life C-30 questionnaire

*p < 0.01 (two-tailed); **p < 0.001 (two-tailed)

Linear regression analysis was performed for men and women. Anxiety, depression, and social support, together with a demographic variable, educational level and clinical variables, functional status and estimated survival, were included in the model as predictors of the dignity score. In men, social support, functional status, and educational level explained 40.3% of the variance in the dignity score (F = 11.913, *p* < 0.001). In women, results indicated that depression, social support, and functional status described 59.6% of the dignity score (F = 22.137, *p* < 0.001), see Table 3. In men, dignity was associated with higher social support, functional status, and higher education level; in women, higher social support, better functional status and lower depression were associated with a higher sense of dignity.

**Table 3:** Linear regression models for dignity and sex

	PPDS-Dignity score				
Predictor	B	***Beta***	***t***	***p***	95%Cl
For men (Constant)	50.357		5.283	**0.001**	31.42 – 69.29
BSI. Anxiety	−0.177	−0.171	−1.365	0.175	−0.43 – 0.08
BSI. Depression	−0.147	−0.115	− 0.973	0.333	− 0.45 – 0.15
DUKE. Social Support	0.515	0.385	4.564	**0.001**	0.29 – 0.73
EORTC-QLQ-C30. Functional Scale	0.084	0.236	2.474	**0.015**	−0.01 – 0.15
Higher educational level	3.234	0.181	2.243	**0.037**	0.19– 6.27
Estimated survival ≥18 months	−0.230	−0.013	− 0.155	0.877	−3.18 – 2.72
*R*^*2*^ adjusted total		0.403			
For women (Constant)	75.336		5.608	**0.001**	48.57 – 96.98
BSI. Anxiety	−0.164	−0.162	−1.119	0.267	−0.45 – 0.13
BSI. Depression	−0.545	−0.380	−2.876	**0.005**	−0.92 – 0.16
DUKE. Social Support	0.434	0.240	3.271	**0.002**	0.17 – 0.69
EORTC-QLQ-C30. Functional Scale	0.107	0.246	2.401	**0.019**	−0.02 – 0.19
Higher educational level	−1.304	−0.060	−0.854	0.396	−4.34– 1.74
Estimated survival ≥18 months	2.661	0.127	1.744	0.085	−0.37 – 5.70
*R*^*2*^ adjusted total		0.610			

*Abbreviations*: *PPDS* Palliative Patient’s Dignity Scale, *BSI* Brief Symptom Inventory, *DUKE* Duke–UNC-11 Functional Social Support Questionnaire, *EORTC-QLQ-C30* European Organization for Research and Treatment of Cancer Quality of Life C-30 questionnaire, *CI* confidence interval. Bold values indicate significance the at 5% level

## Discussion

In this study of patients > 70 years with an advanced cancer, dignity correlated positively with being male, having a higher level of education, longer estimated survival, more social support, less anxiety and depression, and fewer functional limitations.

As for the demographic characteristics of the sample, it must be noted that they are balanced and that most of the subjects included were married and had a primary education. It is important to point out that the data on the dignity scale for women were worse than men. This is in line with previous publications by López-Pina [22] and Kocalevent et al. [23], in which they report that males display higher dignity scores. These sex differences have been associated with women suffering more changes in appearance, more health concerns, more difficulty thinking clearly, and find it harder to have a meaningful spiritual life [8]. These characteristics for females with cancer that influence their dignity should be contemplated as a target for future interventions. Staats et al. [24] conducted a qualitative, descriptive study involving 13 women with terminal cancer who were systematically interviewed. The authors concluded that when physical, as well as emotional and existential needs are met, patients experience their dignity as being preserved. Based on this, it is likely that women’s lower scores on the dignity scale were conditioned by other factors, such as anxiety and/ or depression. Females tend to express more worry and psychological distress than men, and it is possible that the impact of the diagnosis is compounded by concerns surrounding the impact of their illness on their family [25, 26]. However, further studies are needed to elucidate the differences in dignity scores between men and women. In this study, social support, good functional status, greater estimated survival, and less anxiety and depression were associated with higher scores on the dignity scale. Philipp et al. [27] conducted a prospective study and found that loss of interaction with people patients are close to was a strong positive predictor of loss of dignity and often related to feelings of dependency and loss of autonomy. Adequate attachment and social support have also been reported to prevent the onset of depressive symptoms [28, 29]. González-Sáenz de Tejada et al. [30] obtained similar results to ours in a cohort of colon cancer patients. These authors noted that patients who were functionally independent, with better physical and cognitive status and greater social support, had less anxiety and depression. Gray et al. [31] observed that depression correlated with worse physical, social, and cognitive functioning. The relationship between social support, better anxiety and depressive symptom management, and improved well-being and sense of dignity have also been reported in other studies [32–34]. This is corroborated in the regression analysis that demonstrates that 48.8% of the patients’ dignity is explained by the fact that the higher social support, fewer functional limitations, lower anxiety and depression, all lead to patients’ having a greater sense of dignity. It is important to note that with the increase in life expectancy, the elderly population has expanded in recent decades. This population tends to have multiple chronic pathologies, including cancer, hence the importance of improving their needs and quality of life during illness or at the end of life, as several recent studies with these objectives have shown [35–38].

As strengths of this work, in a prospective multicenter series, we have found a direct relationship of lower dignity in the elderly with psychological distress, poor functional status and lower estimated survival, which shows how this construct deteriorates the elderly patient. We have shown that dignity influences how people in need of care are treated in the face of serious illness such as advanced cancer. Other studies have also found that dignity helps patients maintain the highest level of independence and control over their own lives [39]. In our series and in a recent study in people with advanced cancer, patients with poorer functional status and worse estimated survival have low levels of perceived dignity [40, 41]). Hence the importance of developing programs to address the issue of sense of dignity by considering factors that can help preserve dignity such as maintaining good performance status and good social support. Our study has found that addressing a person’s sense of dignity by preserving or enhancing it could be key to their well-being.

### Limitations

The findings of this study must be considered within the context of its limitations. First, the present study was cross-sectional in nature; therefore, it was not possible to determine the directionality of the observed relationships. It is important to develop longitudinal studies to assess the evolution of the variables analyzed at different points in time and how they affect patients’ dignity. Moreover, as we do not have a similar sample of patients ≤70 years of age for comparison, we cannot be sure that the characteristics can be attributed to age or other confounding variables. Second, we used self-report instruments, which can lead to response bias, such as social desirability, memory errors, etc. It is important not to lose sight of the fact that our sample comprises elderly individuals with advanced cancers; consequently, generalization to other ages and tumors can only be undertaken with caution.

## Conclusion

In short, being female, having a lower educational level, shorter estimated survival, depression, anxiety, less social support, and limited functionality are associated with less dignity in elderly patients with advanced cancer.

## Data Availability

All data generated or analyzed during this study are included in this published article.
